# Supplementary figures and images for: Sex-Specific Effects of the Genetic Variant rs10487505 Upstream of leptin in the Development of Obesity
Source: Genes (Basel). 2023 Jan 31;14(2):378. doi: 10.3390/genes14020378 (PMC9956914; doi:10.3390/genes14020378)

(a)

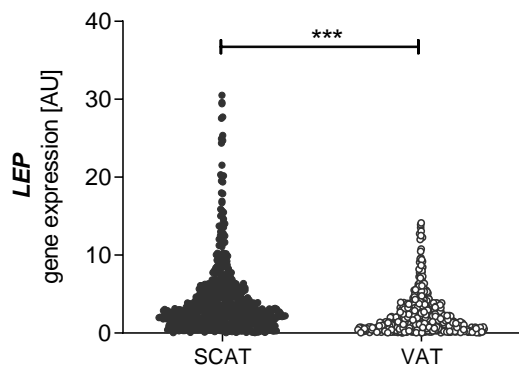

(b)

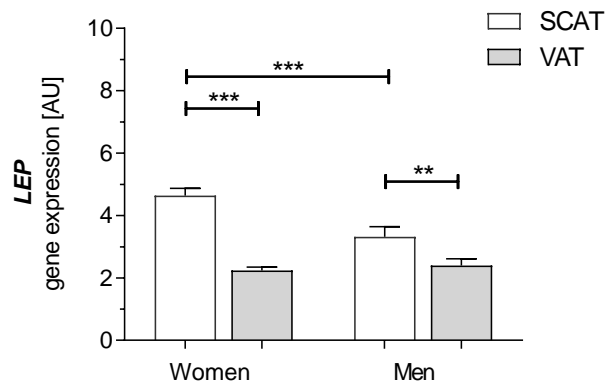

(c)

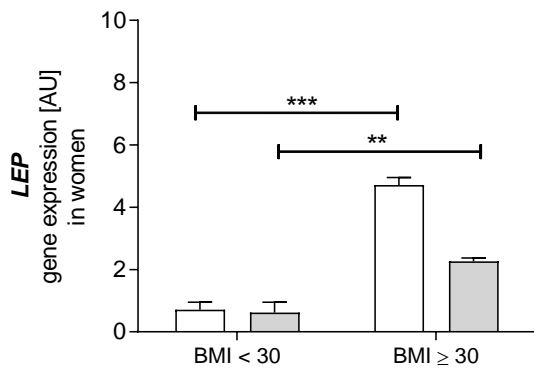

(d)

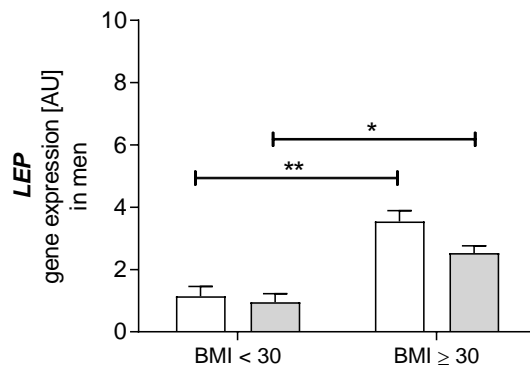

(e)

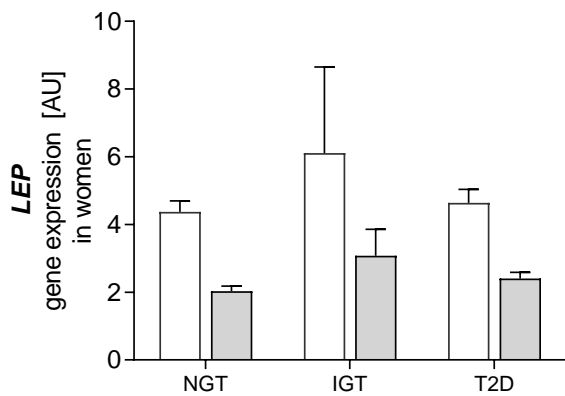

(f)

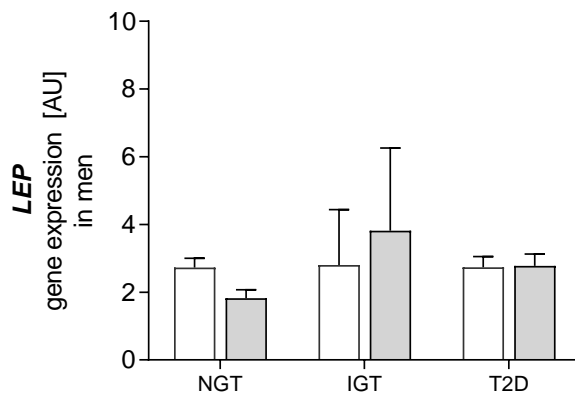

Supplement: Supplementary file 1 [file genes-14-00378-s001.zip › Figure S1.pdf]

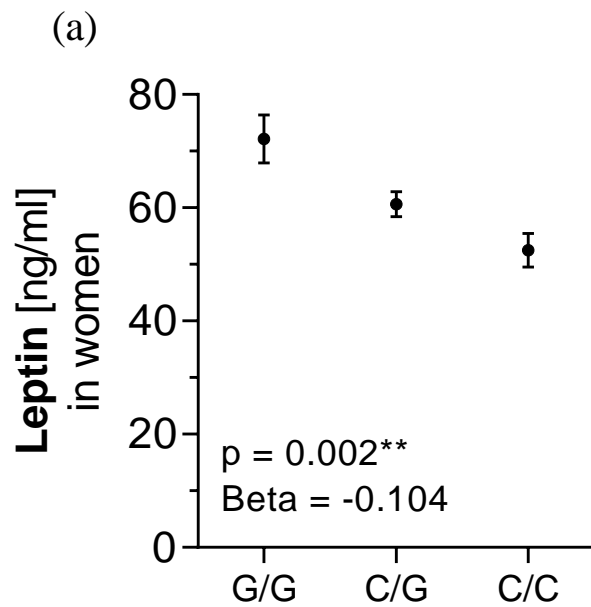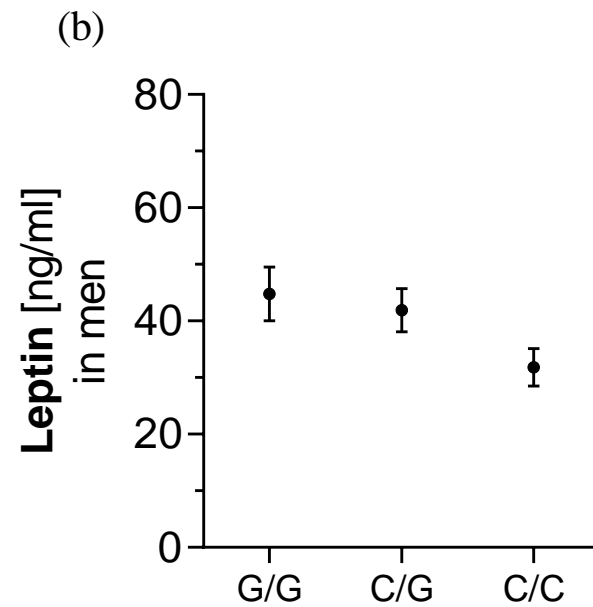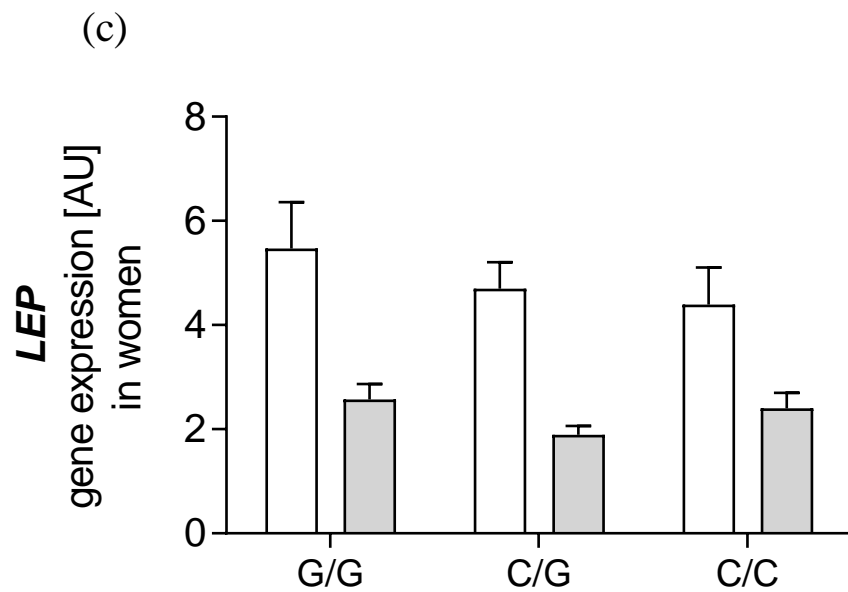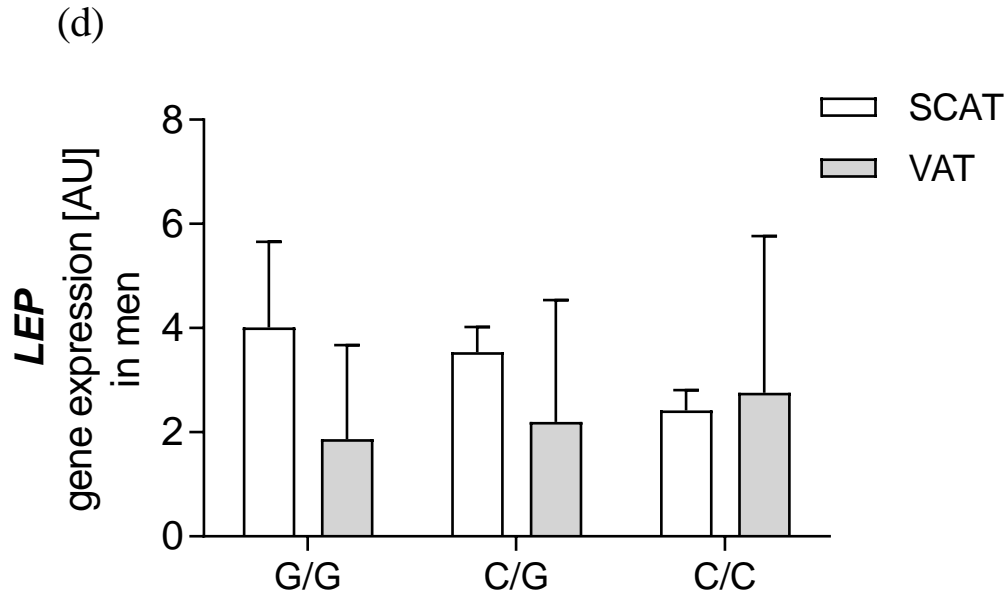

Supplement: Supplementary file 1 [file genes-14-00378-s001.zip › Figure S2.pdf]

(a)

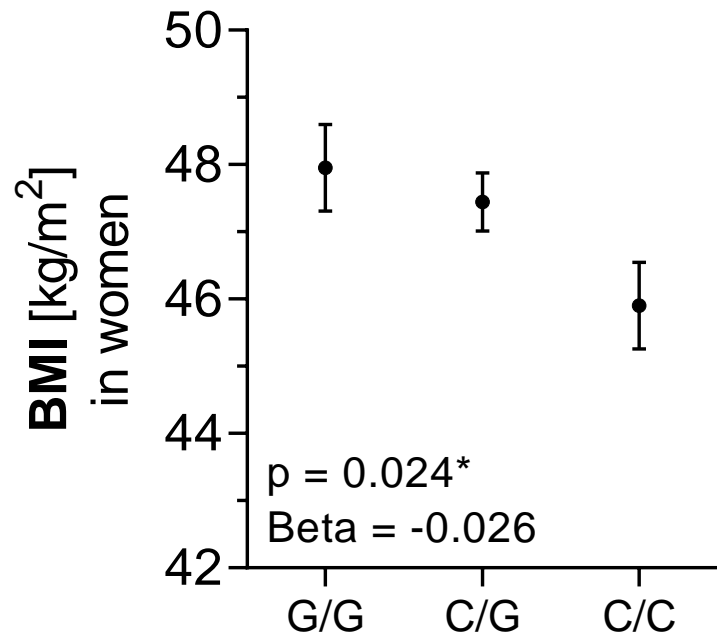

(b)

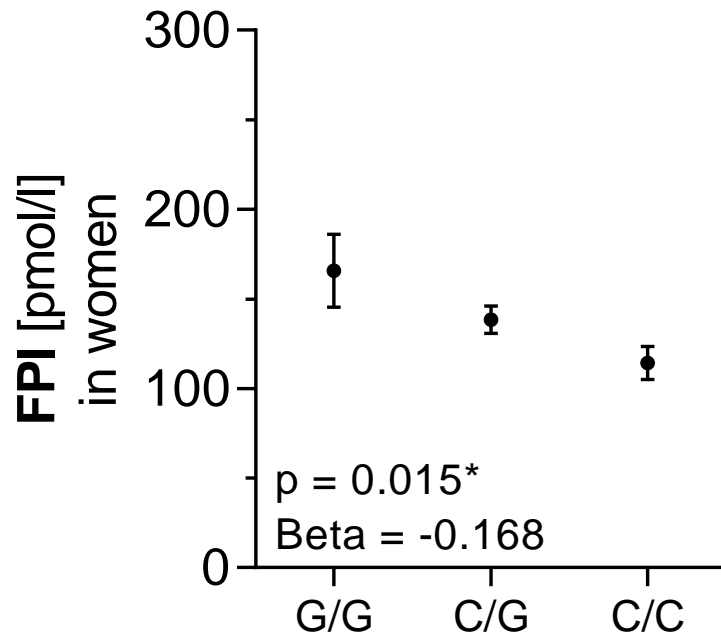

Supplement: Supplementary file 1 [file genes-14-00378-s001.zip › Figure S3.pdf]
